# End-tidal carbon dioxide measurement in preterm infants with low birth weight

**DOI:** 10.1371/journal.pone.0186408

**Published:** 2017-10-17

**Authors:** Hsin-Ju Lin, Ching-Tzu Huang, Hsiu-Feng Hsiao, Ming-Chou Chiang, Mei-Jy Jeng

**Affiliations:** 1 Department of Respiratory Therapy, Chang Gung Memorial Hospital, Taoyuan, Taiwan; 2 Institute of Emergency and Critical Care Medicine, National Yang-Ming University, Taipei, Taiwan; 3 Chang Gung University College of Medicine, Taoyuan, Taiwan; 4 Division of Neonatology, Department of Pediatrics, Chang Gung Memorial Hospital, Taoyuan, Taiwan; 5 Department of Pediatrics, Children’s Medical Center, Taipei Veterans General Hospital, Taipei, Taiwan; University of Oklahoma, UNITED STATES

## Abstract

**Objective:**

There are conflicting data regarding the use of end-tidal carbon dioxide (PetCO_2_) measurement in preterm infants. The aim of this study was to evaluate the effects of different dead space to tidal volume ratios (V_D_/V_T_) on the correlation between PetCO_2_ and arterial carbon dioxide pressure (PaCO_2_) in ventilated preterm infants with respiratory distress syndrome (RDS).

**Methods:**

We enrolled ventilated preterm infants (with assist control mode or synchronous intermittent mandatory mode) with RDS who were treated with surfactant in this prospective study. Simultaneous PetCO_2_ and PaCO_2_ data pairs were obtained from ventilated neonates monitored using mainstream capnography. Data obtained before and after surfactant treatment were also analyzed.

**Results:**

One-hundred and one PetCO_2_ and PaCO_2_ pairs from 34 neonates were analyzed. There was a moderate correlation between PetCO_2_ and PaCO_2_ values (r = 0.603, *P* < 0.01). The correlation was higher in the post-surfactant treatment group (r = 0.786, P < 0.01) than the pre-surfactant treatment group (r = 0.235). The values of PaCO_2_ and PetCO_2_ obtained based on the treatment stage of surfactant therapy were 42.4 ± 8.6 mmHg and 32.6 ± 7.2 mmHg, respectively, in pre-surfactant treatment group, and 37.8 ± 10.3 mmHg and 33.7 ± 9.3 mmHg, respectively, in the post-surfactant treatment group. Furthermore, we found a significant decrease in V_D_/V_T_ in the post-surfactant treatment group when compared to the pre-surfactant treatment group (*P* = 0.003).

**Conclusions:**

V_D_/V_T_ decreased significantly after surfactant therapy and the correlation between PetCO_2_ and PaCO_2_ was higher after surfactant therapy in preterm infants with RDS.

## Introduction

Preterm neonates are vulnerable to lung injuries, especially when they are affected by respiratory distress syndrome (RDS) and mechanically ventilated. Because of rapid changes in lung mechanics after surfactant therapy [1], lung injury and abnormal or fluctuating carbon dioxide levels may occur if the ventilator setting is not adjusted immediately [2]. Thus, continuous monitoring of the adequacy of breathing and oxygenation is necessary. Although pulse oximetry is widely used as a noninvasive method for continuous monitoring [3], oxygen saturation may be normal even if there is inadequate ventilation [4]. Previous studies have indicated that both low and high partial pressures of arterial carbon dioxide (PaCO_2)_ are associated with long-term morbidity in preterm and term infants [5]. In addition, fluctuating PaCO_2_ may lead to lung and brain damage [6, 7], and is associated with retinopathy of prematurity [8].

Mainstream measurement of the partial pressure of end-tidal carbon dioxide (PetCO_2_) is a continuous and noninvasive method to measure blood carbon dioxide tension using with real-time CO_2_ waveforms and numerical values immediately displayed on a monitor [9]. PetCO_2_ has several advantages, such as reduced arterial blood sampling frequency. It also provides a means for the continuous assessment of ventilation without accompanying iatrogenic anemia and is cost-effective [10]. There is a gradient between PetCO_2_ and PaCO_2_ (P(a-et)CO_2_), which can be determined based on the relationship between ventilation (V), which is airflow to the alveoli, and perfusion (Q_A_), which is blood flow to the pulmonary capillaries [11]. On average, the typical V/Q_A_ is 0.8 and PetCO_2_ is normally 2–5 mmHg lower than PaCO_2_, as the mixing volume is diluted in the conducting airways and ends at the alveolar compartment dioxide from the anatomical dead space [12]. V/Q_A_ mismatch occurs due to heterogeneity in the ratio of ventilation to blood flow in different lung units. Areas of the lung that are perfused but not ventilated are said to possess a shunt. Any physiological perturbation that leads to low blood flow levels relative to ventilation in the alveoli increases physiologic dead space and leads to increased P(a-et)CO_2_ [13]. P(a-et)CO_2_ may be caused by shallow breathing, over-inflation of the lung and other cardiac or respiratory pathologies [14]. However, earlier studies examining the effects of changes in dead space to tidal volume ratios (V_D_/V_T_) on PetCO_2_ and PaCO_2_ in newborn infant are scant. The purpose of this study was to evaluate the effects of different V_D_/V_T_ on the correlation between PetCO_2_ and PaCO_2_ in ventilated preterm infants with RDS before and after surfactant therapy. We hypothesized that the difference between PetCO_2_ and PaCO_2_ in ventilated preterm infants with RDS after surfactant therapy will decrease due to the decrease in V_D_/V_T_.

## Materials and methods

### Patient population

This single-center, prospective, non-randomized, consecutive enrollment study was approved by the Institutional Review Board of Chang Gung Memorial Hospital in Taoyuan, Taiwan. Preterm infants with RDS who were admitted to the neonatal intensive care unit (NICU) at Chang Gung Memorial Hospital and treated with survanta (berectant, bovine-derived natural surfactant, AbbVie) between May 2013 and December 2014 were enrolled. Informed consent was obtained from the parents or legal guardians of the patients. Patients with structural cardiopulmonary malformation, those undergoing high-frequency ventilation, and those requiring extracorporeal membrane oxygenation were excluded from the study. The diagnosis of RDS was made based on the classical radiographic appearance, clinical evidence of respiratory distress, laboratory abnormalities due to impaired gas exchange, and the requirement of respiratory support [15]. Surfactant was administered at a dosage of 100 mg/kg, and was divided into 4 quarters following the manufacturer’s recommendation when patients failed to maintain saturations in the normal range when FiO_2_ was >0.4. A second dose of surfactant may be administered if required at least 6 hours after the preceding dose [16]. The patients were ventilated using pressure-limited, time-cycled ventilators in either assist control mode or synchronous intermittent mandatory ventilation mode. The mechanical ventilators (Babylog 8000 Plus, Dräger Medical) were equipped with basic airway graphic monitors and were calibrated following the manufacturer’s recommendations. The initial settings of the ventilator, which were determined using a standard NICU protocol, included a starting respiratory rate of 20 to 40 breaths per minute (bpm) used to maintain a pH of 7.22 to 7.35 and a PaCO_2_ of 40 to 60 mmHg, a peak inspiratory pressure (PIP) of 15 to 25 cmH_2_O, a tidal volume of 4 to 6 ml/kg to produce adequate chest-wall movement, a positive end expiratory pressure (PEEP) of 4 to 6 cmH_2_O to maintain adequate lung expansion, and FiO_2_ adjusted to maintain arterial partial pressure of oxygen (PaO_2_) of 60 to 80mmHg. Infants with very low birth weight (VLBW) whose birth weights were less than 1,500 g were intubated with size 2.5 mm or 3.0 mm endotracheal tubes without cuffs. Non-VLBW (NVLBW) infants whose birth weights were between 1,500 and 2,499 g were intubated using size 3.0 mm or 3.5 mm endotracheal tubes without cuffs.

### Blood sampling

The sampling of arterial blood gas (ABG) was carried out before and 1 hour after surfactant administration, and at 24 hours of age during routine medical care. ABG was measured mainly at the umbilicus arterial catheter, although it was measured at peripheral arteries if the umbilicus arterial catheter was not available. Blood gas determination was performed using a blood gas analyzer (Siemens Rapidlab 248 Blood Gas Analyzer).

### End-tidal carbon dioxide monitoring

PetCO_2_ was continuously monitored using mainstream capnography (Philips M2501A Mainstream Capnography). Since the dead space of the sensors and response times may result in false interpretations of PetCO_2_ readings [17], the sensor was designed for infants with <1 ml of dead space and rise times <60 ms. The infant-type airway adaptor was placed between the endotracheal tube and the Y connection of the ventilator circuit. The capnography device was calibrated according to the manufacturer’s instructions. The sensor for PetCO_2_ was placed prior to blood sampling at each time point. We ensured that the waveform of PetCO_2_ was continuous and steady by measuring expired CO_2_ throughout the ventilator cycle. This allowed us to obtain simultaneous PetCO_2_ and PaCO_2_ measurements. P(a-et)CO_2_ was recorded along with additional data including the mode of ventilation, tidal volumes, PIP, PEEP, total respiratory rate, mean airway pressure (MAP), oxygenation index (FiO_2_ x MAP/PaO_2_), PaO_2_/FiO_2_ ratio, oxygen saturation, blood pressure, and demographic details.

### Dead space to tidal volume ratio (V_D_/V_T_)

V_D_/V_T_ was calculated using the Enghoff modification of the Bohr equation [18]: V_D_/V_T_ = (PaCO2 –PetCO_2_) /PaCO_2_.

### Statistical analysis

Continuous data are expressed as mean ± standard deviation. Statistically significant differences were defined using *P* < 0.05. P(a-et)CO_2_ was assessed using the Bland-Altman technique. The precision of PetCO_2_ and the relationship between PetCO_2_ and PaCO_2_ in various clinical situations was evaluated using Pearson’s correlation coefficients and analyzed using the Statistical Package for the Social Sciences (version 19.0 software). Categorical variables were assessed using chi-square tests. Analyses of variables were performed using independent t tests, while comparisons between the pre-surfactant treatment and post-surfactant treatment groups were carried out using paired t tests. When we compared the parameters according to the treatment stage of surfactant therapy, only the first dose of surfactant was considered.

## Results

One-hundred and one PetCO_2_ and PaCO_2_ pairs were analyzed from 34 neonates who required ventilation due to RDS and were treated with surfactant. The ventilator parameters were calculated according to the first admission sample and were as follows: mean total respiratory rate (53.8 ± 10.5 bpm), mean tidal volume (5.9 ± 0.2 ml), mean ventilation volume per minute (0.4 ± 0.2 L/min.), mean PIP (16.8 ± 2.5 cmH_2_O), mean MAP (9.3 ± 1.2 cmH_2_O), mean PEEP (5.1 ± 0.4 cmH_2_O), and mean FiO_2_ (40.1 ± 10.5%). Sixteen of the infants were NVLBW (mean gestational age 32.3 ± 1.9 weeks and birth weight 1,967 ± 316.5 g). Eighteen infants were VLBW infants (mean gestational age 28.3 ± 1.8 weeks and birth weight 1,084.6 ± 242.6 g). One- hundred and one paired samples (53 from VLBW infants and 48 from NVLBW infants) were used for analysis. The descriptive characteristics of the enrolled patients are depicted in Table 1. There was a significant difference in antenatal corticosteroid use (72.2% vs. 25%, *P* < 0.001) between the VLBW and NVLBW groups. The incidence of bronchopulmonary dysplasia (44.4% vs.25%, *P* = 0.253) and that of patent ductus arteriosus (50% vs.25%, *P* = 0.134) were not different between VLBW and NVLBW groups, as shown in Table 1.

**Table 1:** Descriptive characteristics of the enrolled subjects.

Measure	ALL(N = 34)	VLBW(N = 18)	NVLBW(N = 16)	*p*-value
Male/female, *N*	19/15	6/12	13/3	0.005
Birth weight, *M* ± *SD*, *grams*	1499.9 ± 525.2	1084.6 ± 242.6	1967.2 ± 316.5	<0.001
Gestational age, *M* ± *SD*, *weeks*	30.2 ± 2.7	28.3 ± 1.8	32.3 ± 1.9	<0.001
Antenatal corticosteroid use, *n (%)*	17(50.0)	13(72.2)	4(25.0)	0.010
One dose, *n (%)*	6(17.6)	5(27.8)	0(0)	
Second dose, *n (%)*	10(29.4)	8(44.4)	4(25.0)	
Surfactant dose, *n (%)*	34(100)	18(100)	16(100)	0.021
One dose, *n (%)*	27(79.4)	17(94.4)	10(62.5)	
Second dose, *n (%)*	7(20.6)	1(5.6)	6(37.5)	
**Diagnosis**				
BPD, *n (%)*	12(35.3)	8(44.4)	4(25)	0.253
Mild, *n (%)*	4(11.8)	2(11.1)	2(12.5)	
Moderate, *n (%)*	4(11.8)	2(11.1)	2(12.5)	
Severe, *n (%)*	4(11.8)	4(22.2)	0(0.0)	
PDA, *n (%)*	13(38.2)	9(50.0)	4(25.0)	0.134
Post ligation, *n (%)*	7(20.6)	5(27.8)	2(12.5)	
Ibuprofen treated, *n (%)*	6(17.6)	4(22.2)	2(12.5)	

**Abbreviations**: VLBW, very low birth weight, birth weight < 1500gm; NVLBW, non-VLBW, birth weight≧1500gm; BPD, Bronchopulmonary dysplasia; PDA, Patent ductus arteriosus; P(a-et)CO_2,_ The gradient between PetCO_2_ and PaCO_2;_V_D_/V_T =_ Dead space to tidal volume ratio

*M* ± *SD =* mean ± SD

Analysis of *P* value between VLBW and NVLBW

**Fig 1:**
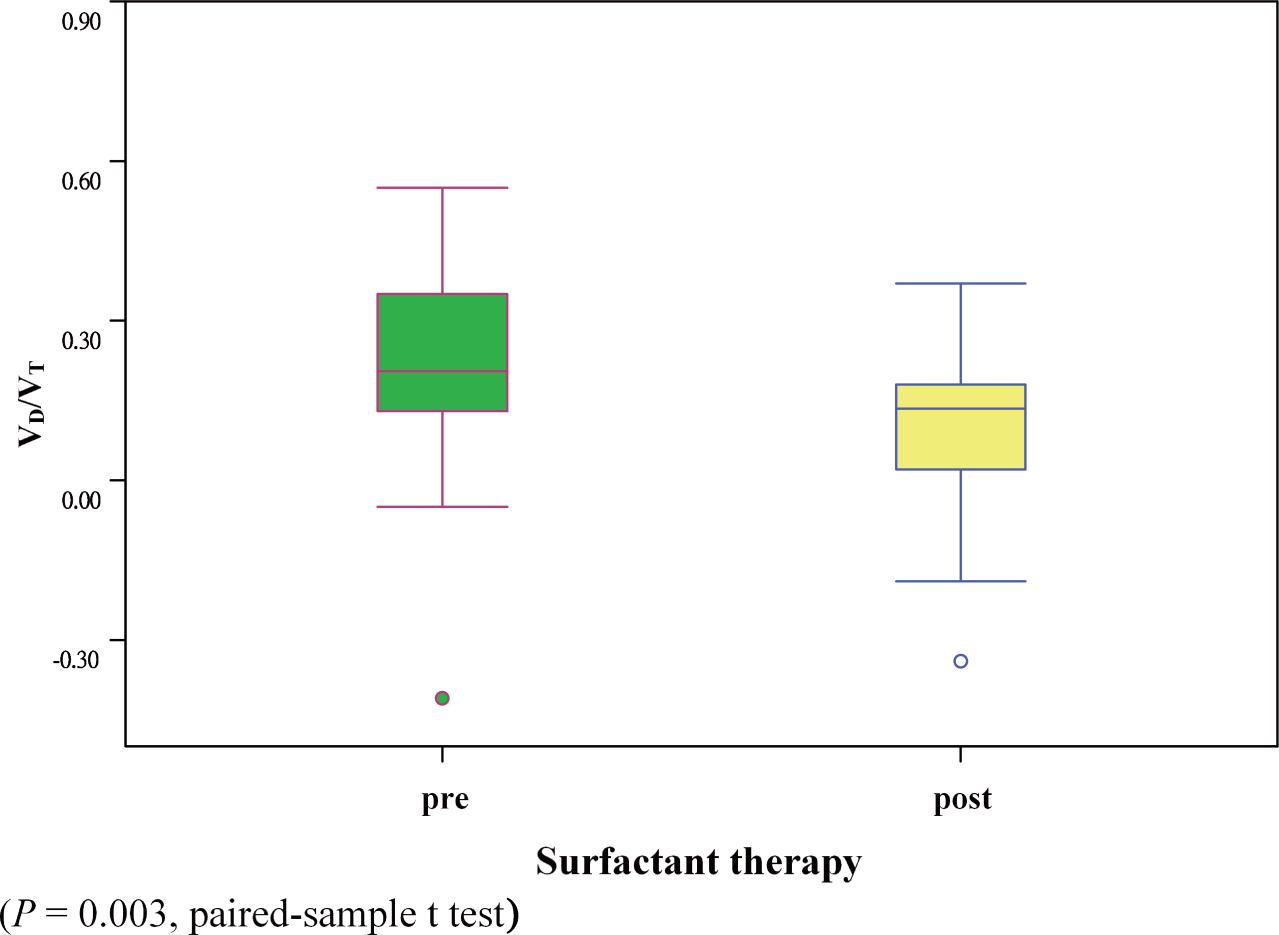
Distribution of V_D_/V_T_ ratio values according to the treatment stage of surfactant therapy (n = 34).

**Fig 2:**
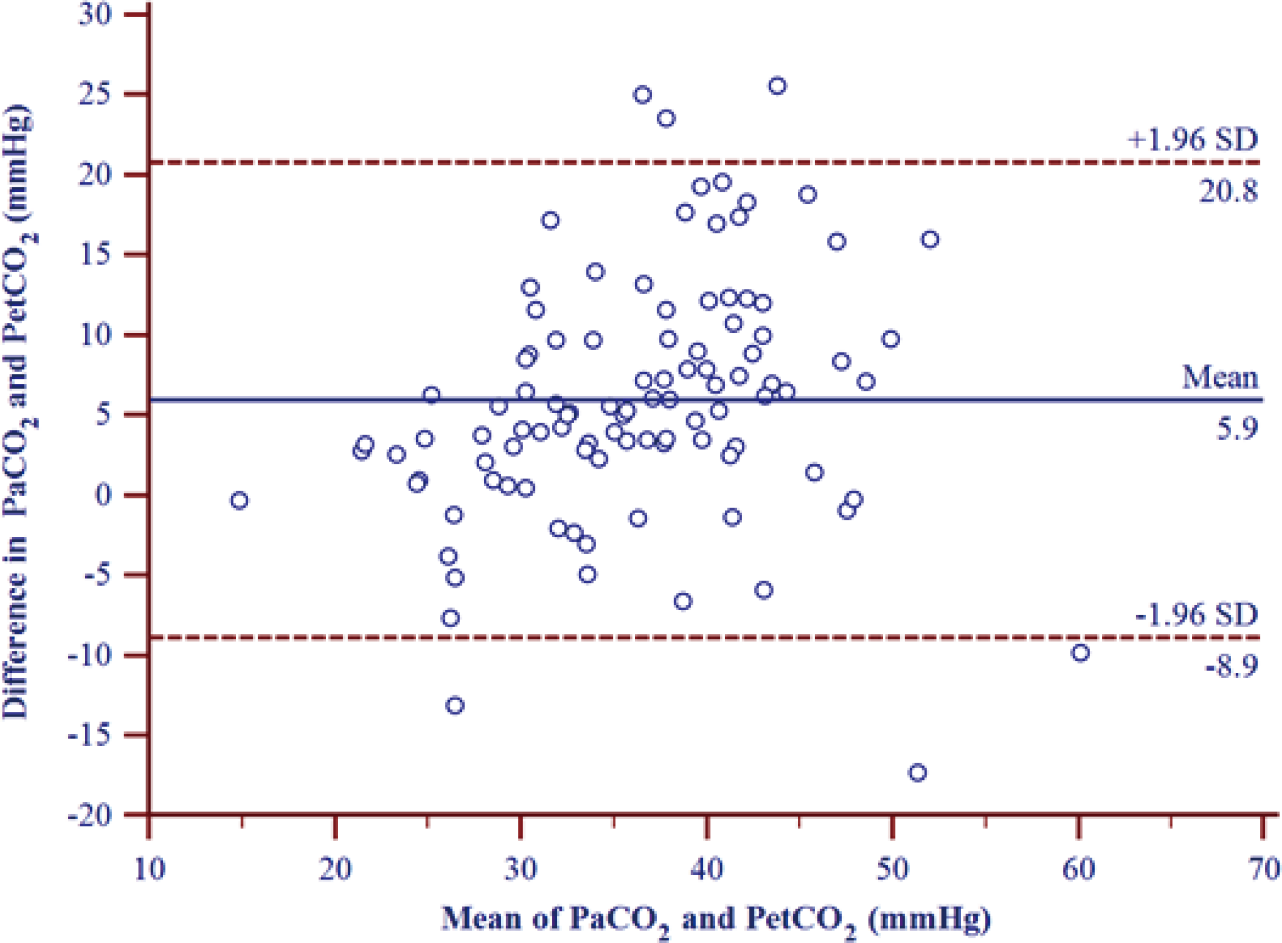
Bland-Altman plot of the difference between the end tidal and arterial CO_2_ levels versus the average of the two simultaneous measurements.

**Fig 3:**
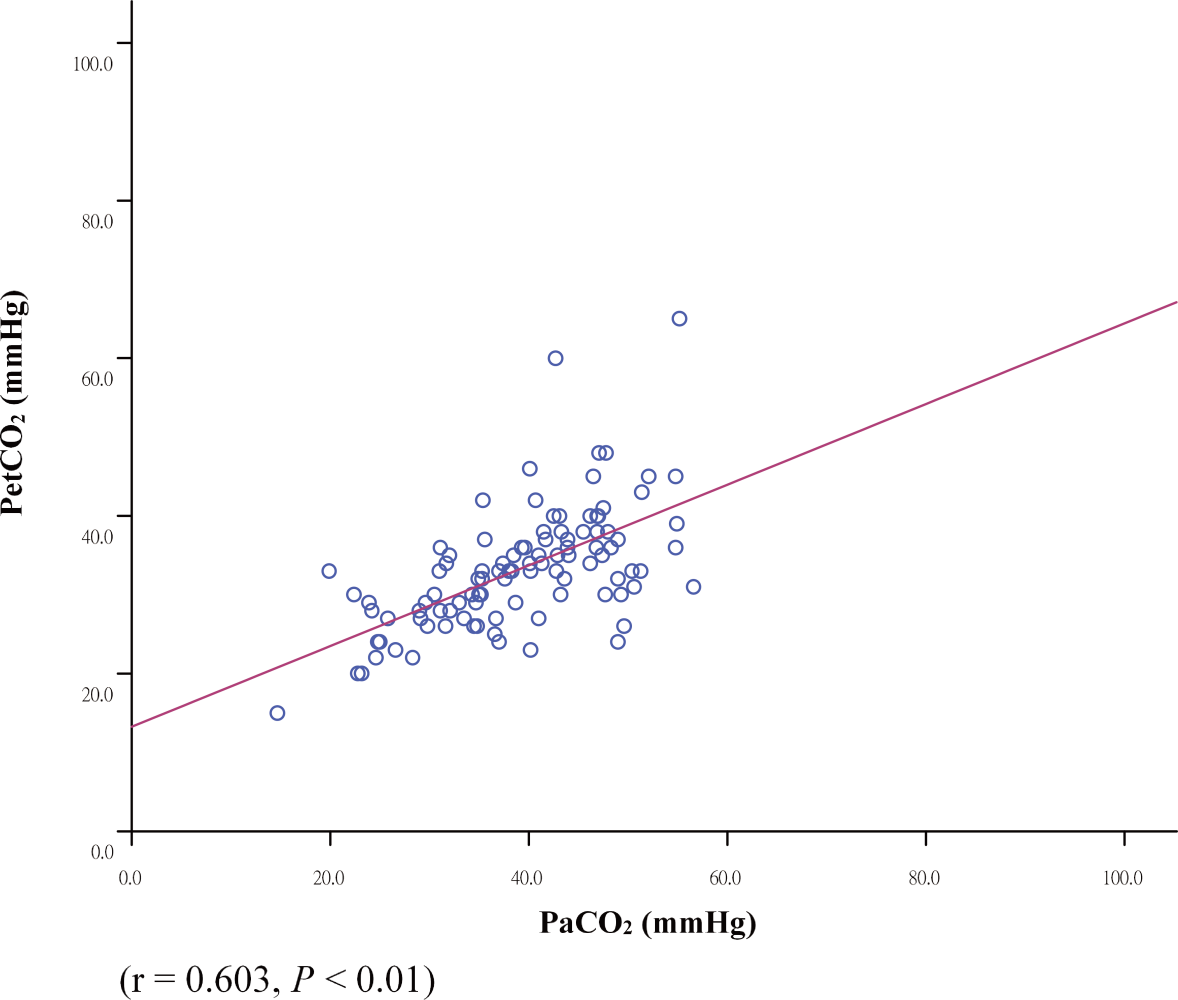
Scattergram plot of the relationship between PaCO_2_-PetCO_2_.

We analyzed difference between patients receiving surfactant before vs. after therapy according to the first dose of surfactant. There was a significant change in V_D_/V_T_, in the post-surfactant treatment group when compared to the pre-surfactant treatment group (*P* = 0.003) (Fig 1). The correlation was higher in the post-surfactant treatment group (r = 0.786, *P* < 0.01) than in the pre-surfactant treatment group (r = 0.235). A significant change in PaCO_2_ (42.4 ± 8.6 mmHg vs. 37.8 ± 10.3 mmHg, *P* = 0.018) and P(a-et)CO_2_ (9.8 ± 9.9 mmHg vs. 4.1 ± 6.5 mmHg, *P* = 0.004) was noted between pre-surfactant and post-surfactant treatment (Table 2). When considering the overall sample data, we found a moderate correlation (r = 0.603, *P* < 0.01) between PetCO_2_ and PaCO_2_. The mean P(a-et)CO_2_ was 5.9 ± 7.6 mmHg. Bland- Altman plots of the comparison of the mean versus the difference in values between PaCO_2_ and PetCO_2_ are shown in Fig 2. A scattergram plot of the PetCO_2_-PaCO_2_ relationship is shown in Fig 3.

**Table 2:** Comparison of parameters accoding to the treatement stage of surfactant therapy.

Measure	pre-surfactant	post-surfactant	p-value
Oxygen index, *M* ± *SD*	6.9±5.2	5.0±3.5	0.055
PaO_2_/FiO_2_ ratio, *M* ± *SD*	190.3±97.4	223.6± 84.5	0.066
PaCO_2_, *M* ± *SD*, *mm Hg*	42.4±8.6	37.8±10.3	0.018
PetCO_2_, *M* ± *SD*, *mm Hg*	32.6±7.2	33.7±9.3	0.439
P(a-et)CO_2_, *M* ± *SD*, *mm Hg*	9.8±9.9	4.1±6.5	0.004

P(a-et)CO_2_ = The gradient between PetCO_2_ and PaCO_2_

## Discussion

In this study, we performed mainstream capnography in infants with RDS who were treated with surfactant. We found that there was moderate correlation, but poor agreement, between PetCO_2_ and PaCO_2_. Some researchers argue that PetCO_2_ may not accurately predict PaCO_2_. Watkins et al. have reported poor correlation between PetCO_2_ and PaCO_2_ in 19 infants with pulmonary disease [19]. Garcia Canto et al. also reported that PetCO_2_ did not have a good correlation with PaCO_2_ in 9 ventilated newborns with severe lung illnesses [20]. More recently, Javier et al. reported that there was larger bias and higher precision between PetCO_2_ and PaCO_2_ than between PaCO_2_ and transcutaneous CO_2_ [21]. This negative result may have been due to the fact that some samples were obtained from babies who were diagnosed with heart failure [21], and that the response time for the PetCO_2_ reading (<150 ms) [19] was much longer than normal (<60 ms). In contrast, Wu et al. observed a higher correlation (r = 0.818, *P* < 0.001) between PetCO_2_ and PaCO_2_ in 61 infants [22]. In 2012, Daniele et al. reported a positive correlation (r = 0.69, *P* < 0.0001) between PetCO_2_ and PaCO_2_ in 45 infants with VLBW [10].

Most previous studies of PetCO_2_ measurements have not considered the severity of lung diseases. Recently, Bhat et al. reported the correlation between PetCO_2_ and PaCO_2_ in a post-surfactant replacement therapy group and concluded that it was more accurate than that in a pre-surfactant replacement therapy group [23]. Similarly, we found a higher correlation between PetCO_2_ and PaCO_2_ in the post-surfactant replacement therapy group than the pre-surfactant therapy group. Furthermore, our results showed that V_D_/V_T_ was decreased significantly after surfactant therapy and that the correlation between PetCO_2_ and PaCO_2_ was higher after surfactant therapy. Based on our finding that the correlation between PetCO_2_ and PaCO_2_ was higher after surfactant therapy, we speculated that our observations may be due to the fact that lung regions with both high and low V_A_/Q can occur simultaneously in patients with RDS [24, 25], while V_D_/V_T_ decreases and the oxygenation index is improved after surfactant therapy [26].

McSwain et al. reported that the correlation between PetCO_2_ and PaCO_2_ improved significantly in patients admitted to the pediatric intensive care unit with lower V_D_/V_T_ (<0.4) [27]. Bindya et al. also reported sidestream PetCO_2_ monitoring provided a more accurate reflection of the PaCO_2_ in patients with lower V_D_/V_T_ (<0.3) [28]. Therefore, PetCO_2_ may be more accurate in post-surfactant treated infants because of the improvement in V_D_/V_T_. Whether sidestream or mainstream PetCO_2_ monitoring is more accurate and suitable for neonates is still controversial [17, 29]. Instead of sidestream PetCO_2_ monitoring, we used mainstream PetCO_2_ monitoring in this study and made similar observation in infants with significant improvements in the PetCO_2_/PaCO_2_ correlation when V_D_/V_T_ was decreased.

This study had some limitations. First, the rate of exposure to antenatal corticosteroids was low in the current study. Only 50% of the patients had received antenatal corticosteroids. However, 72.2% of infants with VLBW received antenatal corticosteroids. Second, we did not measure pulmonary mechanical parameters, such as respiratory resistance and dynamic compliance. Evaluation of these parameters may have been helpful in understanding how physiological abnormalities affect the correlation between PaCO_2_ and PetCO_2_.

## Conclusions

This study was the first to explore the effects of different V_D_/V_T_ values on the correlation between PetCO_2_ and PaCO_2_ in ventilated preterm infants with RDS before and after surfactant therapy. Since ABG analysis is not suitable for the collection of continuous data and the observance of trends, more long-term follow-up studies are required to validate the usefulness of PetCO_2_ for monitoring and evaluating the response to respiratory therapies.
